# Alternative Post-Processing on a CMOS Chip to Fabricate a Planar Microelectrode Array

**DOI:** 10.3390/s111110940

**Published:** 2011-11-22

**Authors:** Francisco López-Huerta, Agustín L. Herrera-May, Johan J. Estrada-López, Carlos Zuñiga-Islas, Blanca Cervantes-Sanchez, Enrique Soto, Blanca S. Soto-Cruz

**Affiliations:** 1 Facultad de Ciencias Físico Matemáticas, Benemérita Universidad Autónoma de Puebla, Av. San Claudio y Río Verde, Col. San Manuel, 72570, Puebla, Puebla, Mexico; 2 Centro de Investigación en Micro y Nanotecnología, Universidad Veracruzana, Calzada Ruiz Cortines 455, 94292, Boca del Río, Veracruz, Mexico; E-Mail: leherrera@uv.mx; 3 Facultad de Matemáticas, Universidad Autónoma de Yucatán, Anillo Periférico Norte, Tablaje Cat., 13613, Col. Chuburná Hidalgo Inn, Merida, Yucatán, Mexico; E-Mail: johanstrd@gmail.com; 4 Instituto Nacional de Astrofísica, Óptica y Electrónica, Calle Luis Enrique Erro 1, 72840, Tonazintla, Puebla, Mexico; E-Mail: czuniga@inaoep.mx; 5 Instituto de Fisiología, Benemérita Universidad Autónoma de Puebla, Av. San Claudio 6301, Col. San Manuel, 72570, Puebla, Puebla, México; E-Mails: blanca.cervantes@gmail.com (B.C.-S.); esoto24@gmail.com (E.S.); 6 Centro de Investigación en Dispositivos Semiconductores, Benemérita Universidad Autónoma de Puebla, Av. 14 Sur y San Claudio, Col. San Manuel, 72570, Puebla, Puebla, Mexico; E-Mail: leriloe@yahoo.it

**Keywords:** CMOS chip, microelectrode array, CMOS post-process, vestibular ganglion neurons

## Abstract

We present an alternative post-processing on a CMOS chip to release a planar microelectrode array (pMEA) integrated with its signal readout circuit, which can be used for monitoring the neuronal activity of vestibular ganglion neurons in newborn Wistar strain rats. This chip is fabricated through a 0.6 μm CMOS standard process and it has 12 pMEA through a 4 × 3 electrodes matrix. The alternative CMOS post-process includes the development of masks to protect the readout circuit and the power supply pads. A wet etching process eliminates the aluminum located on the surface of the p^+^-type silicon. This silicon is used as transducer for recording the neuronal activity and as interface between the readout circuit and neurons. The readout circuit is composed of an amplifier and tunable bandpass filter, which is placed on a 0.015 mm^2^ silicon area. The tunable bandpass filter has a bandwidth of 98 kHz and a common mode rejection ratio (CMRR) of 87 dB. These characteristics of the readout circuit are appropriate for neuronal recording applications.

## 1. Introduction

The complementary metal oxide semiconductor (CMOS) technology can be used to fabricate a planar microelectrode array (pMEA) with high spatial resolution, very good reproducibility and control of their dimensions. The CMOS-based pMEA allows the stimulation and recording of neural activity for cellular networks at the cellular or subcellular level on cultures, *in vitro* or *in vivo* tissue. In the areas of biosensing and neuroscience, the pMEAs have been used for non-invasive multisite extracellular recording [1–3]. The electrical characteristics of the microelectrodes have a strong influence on the reliability and quality of the neural stimulation and recording.

The CMOS technology has allowed the batch fabrication of microdevices integrated with signal readout circuits [4–9], which is called monolithic CMOS-MEMS technology [10,11]. The microstructures designed on a CMOS chip can be released using a post-processing of the chip [12–16], which generally includes some surface or bulk micromachining process. The surface micromachining process can be used to deposit and remove additional layers on the CMOS chip surface, but it is expensive [17,18]. On the order hand, the backside of a silicon wafer can be etched through a bulk micromachining process [19], using either dry or wet etching. Commonly, dry etching is realized through reactive ion etching (RIE) or inductively coupled plasma (ICP). Wet etching uses the etch selectivity between the {111} planes and {100} or {110} planes in aqueous alkaline solutions (e.g., KOH or NaOH). These micromachining processes need the design of a photo-mask set in order to translate the geometric patterns onto a wafer. Generally, most of the post-process steps for either wafer or chip level are complicated. In addition, the post-processing of a chip must protect the electronic circuits during the etching step. In the past, some researchers have released structures using CMOS post-processing [20–23]; although this post-processing did not adequately protect the electronic circuits of the chip [24]. It used a CMOS mask to create a passivation layer in each chip and, next, it completely submerged the chip in the etching solutions. These solutions can penetrate the chip edges, which can damage its electronic components. In addition during dry etching, this passive layer does not correctly protect the component circuits of the chip. In order to overcome these problems, we present an alternative post-process to protect the electronic circuitry and the power supply pads of a CMOS chip, which is etching to release a pMEA. It can be used for monitoring neuronal activity of vestibular ganglion neurons in newborn rats. This post-process step can be easy implemented and adapted to others types of CMOS chips to release their microstructures.

This paper is organized as follows: in Section 2, we describe the readout circuit composed of a bandpass filter and an operational amplifier, and the mechanical frame for handling CMOS chip. Next, in Section 3, an alternative post-processing of the CMOS chip is included. The results of the released pMEA and results of biocompatibility are described in Section 4. Finally, we present the conclusions and future avenues of research in Section 5.

## 2. Design of the Mechanical Frame and Signal Processing Circuit

In biomedicine, systems for recording neuronal activity are essential to diagnose certain diseases. Also, more research on the characteristics of structural organization, dynamics and transient response capability of the neural network in animals such as rats, monkeys, cats, and birds is needed. In addition, the information obtained from the recognition, manipulation and storage of the neuronal signals requires quite high speed signals conditioning and processing systems [25,26]. Finally, it is also necessary that new studies related to biopotential recording systems improve the communication with neurons and simultaneously increase the recording time.

This section presents the design of the signal processing circuit based on CMOS for monitoring the neuronal activity of newborn Wistar strain rats. Furthermore, it shows the design of the mechanical frame that is used to protect the readout circuits and release the pMEA during the micromachining process.

### 2.1. CMOS Integrated Readout Circuit for Monitoring Neuronal Activity

**Figure 1.**
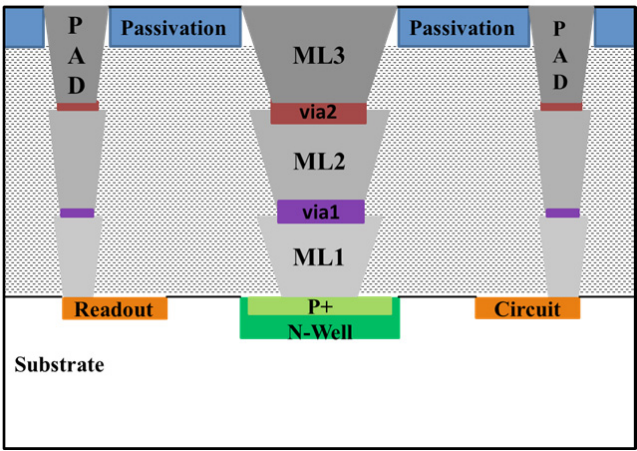
Cross section of the layers employed in the CMOS process.

Our system for monitoring the activity of vestibular ganglion neurons of newborn rats (Wistar strain) is made up by an amplifier, a tunable bandpass filter and a pMEA. The pMEA is fabricated using a standard 0.6 μm triple-metal, double-poly CMOS ON Semiconductor process. The surface of the pMEA has a high p-type doping with a concentration around of 5 × 10^17^ cm^−3^. This array has a surface area of 1 mm^2^ and is placed at the center of a p-type silicon substrate (dimensions of 2.5 × 2.5 mm). The array contains a 4 × 3 microelectrode matrix and each microelectrode has 100 × 100 μm dimensions. Microelectrode separation is equidistant and they are located inside the n-well with a guard ring. This ring is employed to isolate the microelectrodes from the silicon substrate and avoid leakage currents. Figure 1 shows a cross section of the layers used in the CMOS process. In this figure, metal layers (ML1, ML2, and ML3) of aluminum are used as sacrificial material to release the pMEA through the CMOS post-process. The ML1 layer has a thickness of 1.5 μm and both ML2 and ML3 layers have a thickness of 1.6 μm. Each one of these layers is composed of TiN/AlCu/TiN [27]. The initial titaniumtitanium nitride (TiN) layer improves the electromigration robustness and texture [20,28], the aluminum-copper (AlCu) layer is the main conductor, and the top TiN layer is antireflection coating (ARC) [28]. Stacked layers (ML1, via1, ML2, via2, and ML3) are placed above the surface area of the pMEA. The connections of between the metallic layers are made with tungsten (W) vias through via1 and via2 to satisfy the design rules of the commercial CMOS process. These layers are etched without damaging the readout circuit and power supply pads.

**Figure 2.**
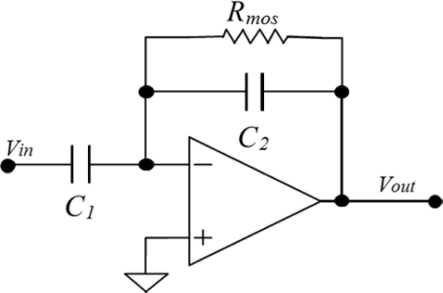
Readout circuit for recording the neuronal activity.

Figure 2 shows the readout circuit used for recording the neuronal activity, which is employed in applications of biological signals with bandwidth of 98 kHz. Its low cutoff frequency (*f_LC_*) is determined by:

(1) $$f_{\mathrm{LC}}=\frac{1}{2\pi R_{\mathrm{mos}}C_{2}}$$

where *R_mos_* is the resistance emulated through the transmission gate and operated in the subthreshold region [26], which has a minimum value of 1.5 GΩ. The capacitors *C_1_* and *C_2_* are of 10 and 0.1 pF, respectively.

**Figure 3.**
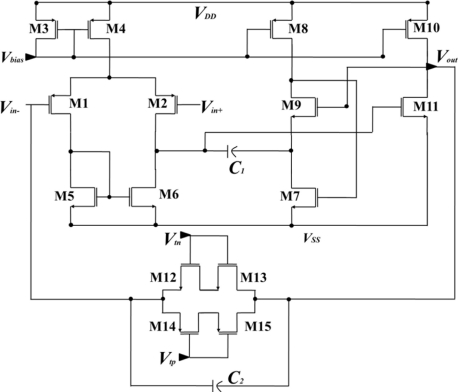
Schematic of the tunable bandpass filter used for recording neuronal activity.

In order to integrate both the pMEA and the readout circuit, we used the geometric pattern editor of Cadence^®^ Virtuoso. The design of the readout circuit has low power consumption (close to 360 μW). The readout circuit is formed of an analog amplifier and a tunable bandpass filter (see Figure 3), which are designed with a voltage gain of 100. The amplifier has a first stage integrated by differential-pair transistors M1–M2 and a polarization through transistors M5 and M6. The biasing of the circuit is made through a current mirror that is composed of transistors M3 and M4. In order to improve the stability of this circuit, the compensation is implemented by a flip voltage follower, which is made with the transistors M7 and M9 [29], as well as a capacitor *C_1_*. The final stage consists of transistors M10 and M11 that form a buffer. The first and last stages are coupled by transistors M1, M12, M13, and M14, which integrate the transmission gate. It is used to implement the tunable bandpass filter and capacitor *C_2_*. During the realization of neurological experiments, it is important to have the ability to simultaneously record local field and neurons. It is desirable to obtain an integrated readout circuit with a tunable low-frequency cutoff [30]. The precise tuning of the low-frequency cutoff of the preamplifier to the lowest frequency of interest can remove undesirable out-of-band noise and yields the fastest settling time for a given application. The subthreshold-biased MOSFET is used to implement *R_mos_* in the readout circuit showed in Figure 2. The exponential dependence of the conductance on threshold voltage and other process parameters that make the low-frequency cutoff highly variable can be trimmed in this design by adjusting the tunable voltage. Table 1 shows the channel length and width ratios of all MOS transistors obtained for the proposed design in this work.

### 2.2. Mechanical Frame

**Figure 4.**
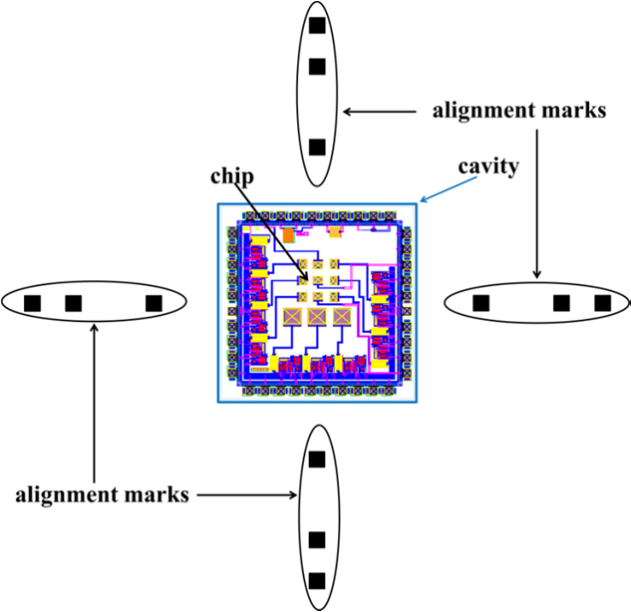
Layout of the mechanical frame employed for handling the CMOS chip.

The standard design of our integrated circuits have a surface area of 2.5 × 2.5 mm and are returned by the commercial foundry on silicon die with a surface area approximately 3.51 × 2.5 mm [31]. These data are used to design the photo-mask set, which is used to fabricate the mechanical frame. The design of the masks was made using the INAOE 1:1 technology file. This step is critical, because it must be scaled properly to the chip and the mechanical frame dimensions. The fabrication process of this mechanical structure employs a Pyrex glass substrate [32] (dimensions 76.2 × 50.8 × 2 mm) to form the cavity; furthermore, it uses 12 external alignment marks, as shown in Figure 4.

The process of transferring the geometric pattern is made by the conventional technique of photolithography. Generally, the CMOS-post process uses additional sacrificial layers to form the microestructures, but this can generate residual stress, stress gradients, and curling. In order to overcome these problems, we avoid these sacrificial layers and propose a mechanical frame. The fabrication process of this frame includes the following steps: (1) mechanical support with aluminum to form the square alignment marks and (2) wet etching to release the cavity of the Pyrex glass.

In order to etch the aluminum, we used a solution composed of phosphoric acid (H_3_PO_4_) with a concentration of 0.12 mol L^−1^ (also written as 0.12 M), glacial acetic acid (CH_3_COOH) with a concentration of 3.84 M, and nitride acid (HNO_3_) with a concentration of 0.71 M using a relation by volume of 75:22:3 for 15 minutes at 50 °C. On the order hand, the Pyrex glass was etched using a solution composed of hydrofluoric acid (HF) with a concentration of 51.04 M and hydrochloric acid (HCl) with a concentration of 2.93 M. The volume mixing ratio of HF and HCl was of 10:1 for 10 min at room temperature.

The first step of our CMOS post-processing includes the design of the mechanical frame, which allows the handling of the chip to eliminate the aluminum located on the surface of the p^+^-type silicon.

### 2.3. Metallic Mask

**Figure 5.**
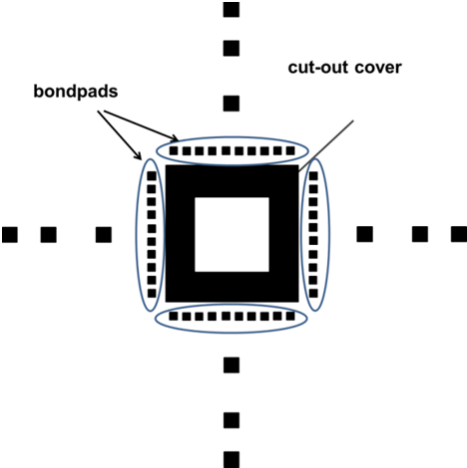
Geometric pattern used in the metallic mask.

A metallic mask is used to protect the chip during the etching process. It is based on a thin steel sheet (type 302 with 76 μm of thickness) of Precision Branded^®^ Stainless Steel [33]. The geometric pattern of the metallic mask has twelve alignment marks (three per edge). The marks are square in shape (200 × 200 μm) and located 0.5 mm, 1.5 mm and 2 mm from the chip edges (see Figure 5). These geometric tolerances are used to ensure a better alignment between the metallic mask and mechanical frame. The geometric patterns of the metallic mask are etched using a mixture of H_3_PO_4_ (concentration of 0.04 M), HNO_3_ (concentration of 4.36 M), CH_3_COOH (concentration of 5.95 M), and H_2_O (concentration of 3.95 M) with a volume mixing ratio of 1:1:1:1 for 3 min at room temperature.

## 3. Description of the CMOS Post-Process

**Figure 6.**
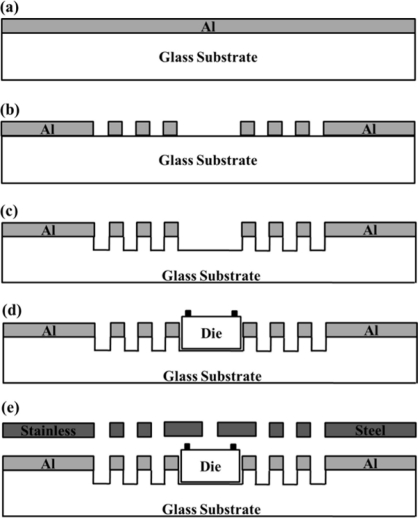
**(a–e)** Steps of the post-processing on a CMOS chip to fabricate a pMEA.

**Figure 7.**
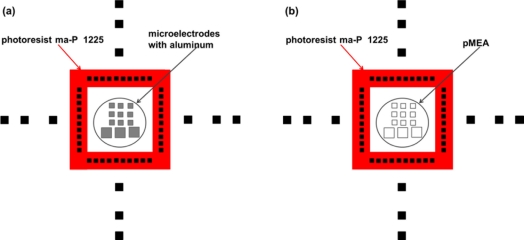
Positive photoresist deposited over the bondpads and microelectrodes with **(a)** aluminum and **(b)** without aluminum.

The CMOS post-process starts with the deposition of an aluminum layer (1.5 μm thickness) over a Pyrex glass substrate, as shown in Figure 6(a). The geometric patterns of the mechanical frame are transferred to the aluminum layer to etch the alignment marks. Next, the Pyrex glass substrate is etched to form a cavity where the CMOS chip is collocated, as shown in Figure 6(c). The chip is glued in the central cavity of the mechanical frame using a temporary mounting wax (QuickStick 135) [34]. This wax is heated to its melting point (71 °C) and applied in the cavity of the mechanical frame. In the following step, the mechanical frame is aligned with the metallic mask, as shown in Figure 6(e). They are used for handling the chip and to deposit a positive photoresist (ma-P 1225) [35] over the bondpads of the chip, as shown in Figure 7(a). This photoresist is used as protection layer during the water etching. Figure 7(b) shows the release of the aluminum to obtain the pMEA. This photoresist is rotated to high velocity (3,000 rpm) using a spin coating for 3 minutes to obtain thin films with thickness less than 2 μm. In order to obtain a polymerized photoresist, the chip with the metallic mask and the mechanical frame is placed on a hot plate at 100 °C for 15 min. After, a wet etching process eliminates the aluminum deposited above the surface of the p^+^-type silicon. This etching process is made with a chemical solution composed of H_3_PO_4_ (0.12 M), CH_3_COOH (3.84 M), and HNO_3_ (0.71 M) with proportions by volume of 75:22:3, which is applied for 20 minutes at 50 °C. Then, the chip is removed from the chemical solution and cleaned with deionized water through an ultrasonic cleaner for 5 min at room temperature. The positive photoresist is removed using acetone for 2 min at room temperature. Later, the wax is heated to 71 °C to separate the chip from the cavity in the mechanical frame. Finally, the impurities are eliminated using acetone and deionized water.

The following section indicated the results of the CMOS post-process proposed in this work.

## 4. Results of the CMOS Post-Process

### 4.1. Released pMEA

**Figure 8.**
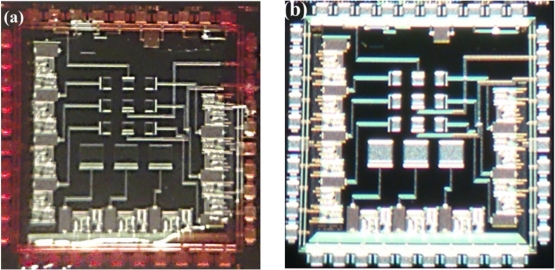
Microphotograph of a single die **(a)** before and **(b)** after CMOS post-process.

In order to check the results of our alternative post-processing on a CMOS chip, it is necessary to revise the release of the p^+^-type silicon of the pMEA. Figure 8(a,b) shows the microphotographs of the chip before and after the CMOS post-process. In this microphotograph, the zones of silver color indicate the bondpads and stacked layers on the surface area of the pMEA.

**Figure 9.**
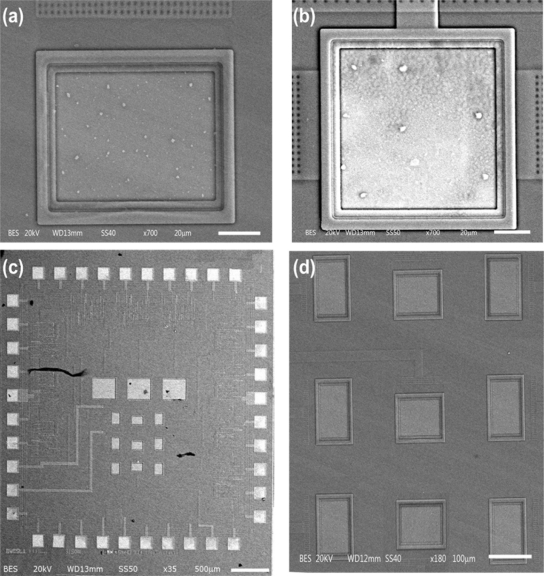
SEM images of the CMOS post-process, which were obtained **(a)** microelectrode of the pMEA, **(b)** power supply pad without damage, **(c)** chip after of the CMOS postprocess and **(d)** the released pMEA through the CMOS post-process.

The active areas of p^+^-type silicon did not show structural damages, as shown in Figure 9(a), where the residues on the surface microelectrode of the pMEA are products of alloy composed silicon and aluminum. This product does not represent an issue for monitoring the neuronal activity. In addition, the bondpads were etching without present damages, as shown in Figure 9(b). The released pMEA through the CMOS post-process was successfully obtained and did not present any structural damages. Figure 9(c) shows the complete chip after of the CMOS post-process. In this Figure, the pMEA and bondpads do no present any cracks. Figure 9(d) shows the pMEA released as a result of the CMOS post-process. The SEM images were recorded with acceleration voltage of 20 kV at high vacuum (HV) using JEOL SEM model JSM-5610LV (Hitachi, Japan). The chip was placed in specimen stub using double side adhesive carbon tape.

**Figure 10.**
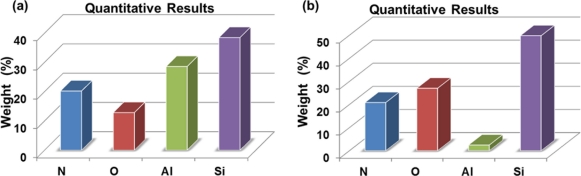
Results of the molecular weight of the materials placed on the surface areas of the pMEA, which were obtained **(a)** before and **(b)** after of the CMOS post-process.

**Figure 11.**
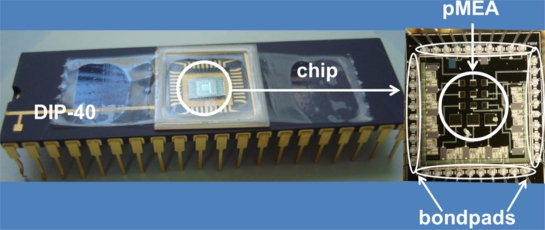
Microphotograph of chip packaged in a DIP-40.

In order to verify the correct release of the pMEA, we measured the chemical composition in the surface areas of the pMEA before and after of the CMOS post-process. For this, we used the energy dispersive spectroscopy (EDS) facility available with the SEM. The chemical composition measurements of the pMEA show the molecular weight of its materials. Figure 10 indicates the results of the EDS of the pMEA before and after the CMOS post-process. After the CMOS post-process, the molecular weight of the aluminum electrodes decreases approximately 25%. It is due to the etching of the aluminum layers on the surface area of the pMEA. Finally, the chip is packaged in a DIP-40 (dual in line package), as shown in Figure 11.

### 4.2. Biocompatibility of pMEA

The pMEA can be used for monitoring the activity of either slices of brain tissues or neurons grown on the chip. In order to check the biocompability of the surface of the p^+^-type silicon (pMEA), we made primary cultures of vestibular ganglion neurons to test the biocompatibility and electrical performance characteristics of the readout electric circuit of the CMOS chip. For neuron extraction culture and recording, we used previously published procedures [36,37]. Animal care and procedures were in accordance with the National Institutes of Health Guide for the Care and Use of Laboratory Animals as outlined in the “Guide to the Care and Use of Laboratory Animals” issued by the National Academy of Sciences. Animals were supplied by the “Claude Bernard” animal house of the Autonomous University of Puebla. Briefly, Wistar rats (n = 6) of postnatal day 7–10 age were anesthetized with servofluorane and killed by decapitation. The head was cleaned with 70% ethanol, the inferior maxillar was removed and the cranium immersed in L-15 culture medium (Gibco, Grand Island, NY, USA). Next, the upper part of the skull and brain were extracted. The otic capsule and the vestibular ganglia were identified with a stereoscopic microscope (Nikon, Tokyo, Japan). The vestibular ganglia were dissected and enzymatically dissociated using a combination of 1.25 mg/mL porcine trypsin and 1.25 mg/mL collagenase-IA dissolved in L-15 culture medium for 30 min at 37 °C. The ganglia were rinsed with fresh culture medium, triturated with a fire-polished Pasteur pipette, and centrifuged for 5 min at 4,000 rpm. In the following procedure, we discarded the supernatant. This procedure was repeated three times. The isolated neurons were plated in the p^+^-type silicon (pMEA) placed in Nunclon petri dishes (Nunc, Roskilde, Denmark), and cultured in a 95% air-5% CO_2_–humidified incubator at 37 °C for 18–24 h. The cells cultured on the substrate were maintained in L-15 media supplemented with 10% BFS, 500 IU penicillin, 25 μg/mL fungizone, 15.7 mM NaHCO_3_, 15.8 mM HEPES, and the pH adjusted to 7.7 using NaOH. A pH of 7.7 was employed to allow it to reach a pH of 7.4 after 30 min in a CO_2_ incubator. For the electrophysiological recording, the culture dish was mounted on the stage of an inverted phase-contrast microscope (TMS, Nikon).

Voltage-clamp recording and data analysis of afferent neurons were done at room temperature (23 to 25 °C) and cells were perfused with extracellular solution (in mM: NaCl 140, KCl 5.4, CaCl_2_ 1.8, MgCl_2_ 1.2, HEPES 10, pH 7.4). Ionic currents from the neurons were recorded using the patch-clamp technique in the whole-cell voltage-clamp configuration. Patch-clamp pipettes were fabricated from 1.2-mm outside diameter borosilicate-glass capillary tubing (WPI, Sarasota, FL, USA) using a horizontal Flaming Brown micropipette puller (P80/PC Sutter Instruments, San Rafael, CA, USA). Patch-pipette resistance was 1 to 3 MΩ when filled with the intracellular solution (in mM: NaCl 10, KCl 135, CaCl_2_ 0.134, HEPES 5, EGTA 10, pH 7.2). Cells were also recorded in current-clamp conditions to study their membrane potential (Vm) and their responses to current injection from −0.5 to 1 nA in 0.1 nA steps generated using pClamp software.

Data were recorded with an Axopatch 200B amplifier (Molecular Devices, Union City, CA, USA), the output of which was led to a 12-bit digital-analog converter (Digidata 1200, Molecular Devices) controlled by pClamp 9.0 software (Molecular Devices). The membrane capacitance (Cm) and series resistance (Rs) (≈80%) were electronically compensated. Data were low-pass filtered at 2 kHz and sampled at 5 kHz. Clampfit 9.0 (Molecular Devices) and Origin 6.0 (Microcal Software, Inc., Northampton, MA, USA) were used for the analysis and fitting of recordings.

To demonstrate that cells grow on the chip were neurons, the expression of 160 KDa neurofilaments (NF-160) (specific staining for neurons) was used. In addition, monoclonal antibodies (IgG1) against the NF-160 (Sigma Chemicals, St. Louis, MO, USA) were used. For this the chip with cultured afferent neurons were fixed for 30 min with 4% paraformaldehyde in phosphate buffer solution (PBS), washed with 0.1 M PBS, pH 7.4, incubated with fetal goat serum added with 0.03% Triton X-100 and 0.2% bovine serum albumin (BSA) for 30 min. The preparations were then incubated in a humid chamber with the antibodies against NF-160 (diluted 1/40 in 0.1 M PBS pH 7.4 with Triton X-100 at 0.03% and 0.2% BSA) for 1 h at 37 °C and 1 hr at room temperature (about 22 °C). Specific labeling was obtained by incubating with an Alexa Fluor 568 F(ab’)_2_ fragment of goat anti-mouse IgG, diluted 1/200 (obtained from Invitrogen, Grand Island, NY, USA) for 2 h at room temperature. In all cases controls were performed in which the first antibody was omitted. Observation was done using a laser scanning confocal microscope (LSM 5 Pascal AxiosKop2MOT, Carl Zeiss, Jena, Germany). Image processing and labeling were done using Zeiss LSM Image Examiner.

**Figure 12.**
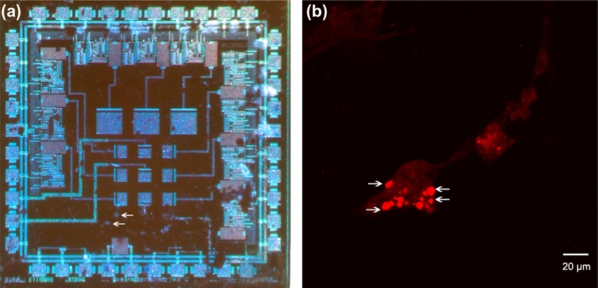
Neurons of vestibular ganglion in newborn rats cultured on the pMEA surface. **(a)** Microphotograph of a chip upon which neurons had been cultured (arrows) and **(b)** antineurofilament staining of the cells cultured on the chip. Specific immunostaining with antibodies against the NF-160 demonstrate that cells in the culture are neurons.

**Figure 13.**
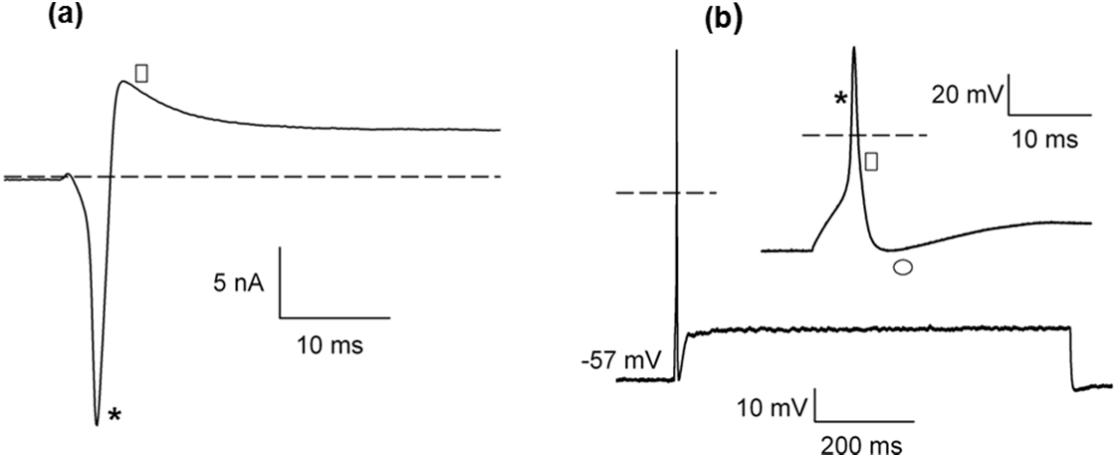
Electrophysiological recording of neurons. **(a)** Voltage clamp recording shows an initial rapid inward current (*) followed by a slowly inactivating outward current (⎕) (dashed line represents zero nA). In **(b)** recording of the voltage response of a neuron to a current pulse of 600 pA. A typical action potential is discharged. In the inset, a time expansion of the action potential shows its typical characteristics with a fast rising phase (*) that reaches a peak value of about 40 mV. This is followed by a repolarizing phase (⎕) and post-potential hyperpolarization (R) that slowly decays (the dashed line represents 0 mV).

For the experiments the neurons were cultured upon the pMEA surface, as shown in Figure 12(a). Evidence that cells growing in the chip surface were already neurons was obtained by staining with anti NF-160 Figure 12(b). The neurons grown on the surface of the p^+^-type silicon were subject to electrophysiological recording to demonstrate that they were already alive and capable of generating membrane ionic currents and action potentials. Voltage clamp recordings shown that these neurons display typical ionic currents (compare with those obtained in previous works [36,37]), including a fast inward current at the beginning of voltage clamp pulse (most probably a Na^+^ current) and ensuing outward currents (corresponding to a set of K^+^ currents), as shown in Figure 13(a). In current clamp mode experiments, the membrane voltage of the cells was about −60 mV and under depolarizing current pulse injection the neurons were capable of firing typical action potentials similar to those previously described in standard substrate culture, as shown in Figure 13(b).

### 4.3. Electrical Performance Characteristics of the Readout Electric Circuit of the CMOS Chip

**Figure 14.**
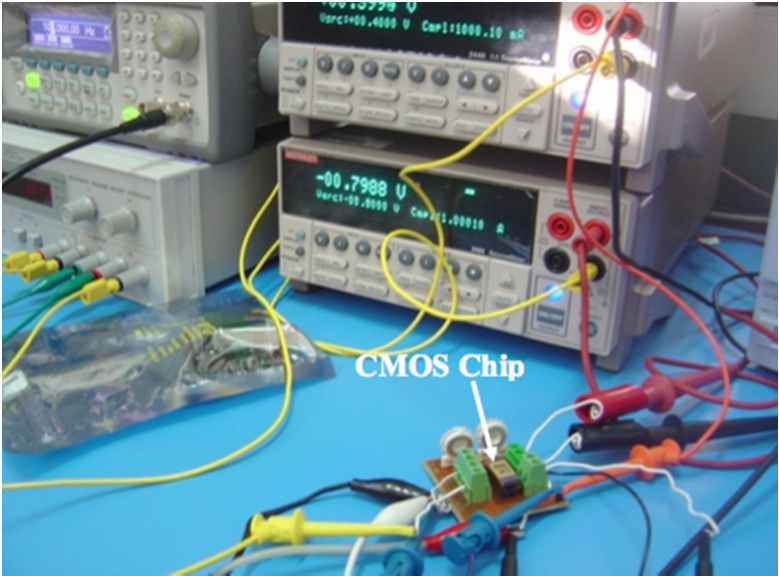
Experimental setup used to obtain electrical performance characteristics of the readout electric circuit of the CMOS chip.

**Figure 15.**
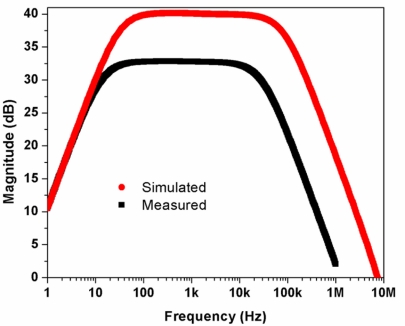
Frequency response of the readout electrical circuit of the CMOS chip.

**Figure 16.**
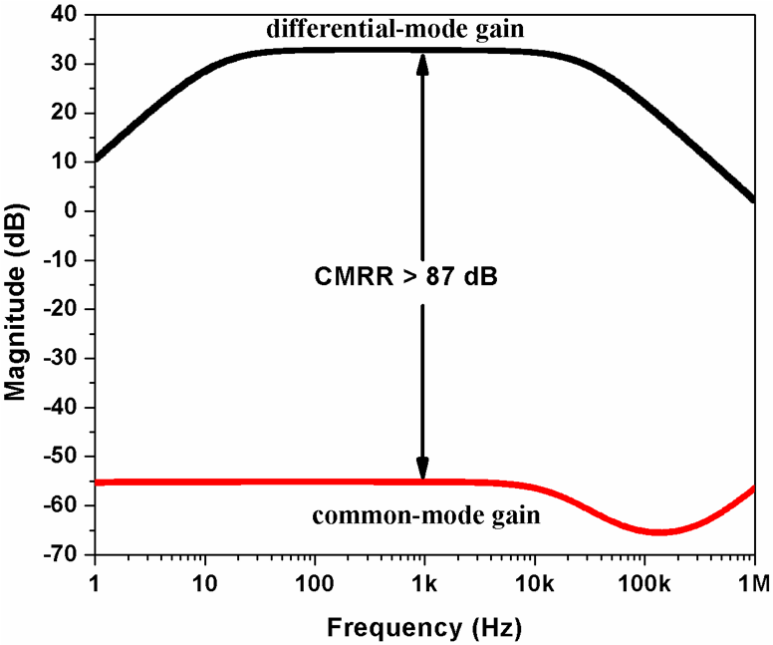
Common mode rejection ratio (CMRR) measured in the readout electrical circuit of the CMOS chip.

The preliminary test of the electrical performance characteristics of the readout electric circuit of the CMOS chip was made using an arbitrary waveform generator (Agilent^®^ 33220A), a digital oscilloscope (Lecroy^®^ Wave Surfer), and two current sources (Keithley^®^ 2400 and 2440), as shown in Figure 14. A sinusoidal excitation signal of 50 μVpp at 1 kHz is supplied to the readout electric circuit and its output signal is amplified more than 100 times (40 dB), as shown Figure 15. In order to measure the low cutoff frequency of the readout electrical circuit, we applied a frequency sweep for its input signal from 5 Hz to 10 kHz. The common mode rejection ratio (CMRR) measured in the readout electrical circuit was 87 db at 20 kHz, as shown in Figure 16.

Table 2 indicates a comparison of the electrical characteristics of our readout circuits for recording the neuronal activity with respect to those reported in the literature. Our readout circuit has a chip area smaller than those reported by several researchers [26,30,38]. In addition, the CMRR of our readout circuit is higher than those obtained in other works [26,38].

The results of these preliminary tests will be used in future research to optimize the characteristics of the pMEA and readout electrical circuit of the CMOS chip.

## 5. Conclusions

An alternative post-processing on a CMOS chip was developed to release a pMEA without damaging the readout circuits and the bondpads. This CMOS post-process did not require the deposition of additional materials (e.g., metal and oxide layers) on the chip. In addition, it can be easy employed with a low cost. The design of a mechanical frame is included for handling the chip during the etching process. The released pMEA did not present either structural cracks or geometric defects. This CMOS post-process can be implemented in other integrated circuit fabrication technologies. The biocompatibility shows that isolated rat neurons may be maintained in culture on the surface of the p^+^-type silicon, and that these neurons maintain their typical electrical behavior. Future work will include the etching of the chip backside to obtain a thin membrane (5 μm thickness) that supports the pMEA. This can help with recordings of the neuronal activity.

## Figures and Tables

**Table 1.** Aspect ratio (length/width) of the transistors for the proposed design.

**Devices**	***W*/*L* (μm)**

M1–M2	60/4.8
M3–M4	40.05/2.4
M5–M6	19.95/5.4
M7	10.05/3
M8	40.05/2.4
M9	40.05/1.5
M10	150/2.4
M11	75/2.4
M12–M15	10.05/1.5

**Table 2.** Electrical characteristics of some readout circuits for recording the neuronal activity.

**Parameter**	**Wattanapanitch *et al*. [26]**	**Olsson *et al*. [30]**	**Majidzadeh *et al*. [38]**	**Our work**

Technology	0.6 μm	3 μm	0.18 μm	0.6 μm
Area (mm^2^)	0.16	0.177	0.65	0.014
Gain (dB)	40.85	38	39.4	40
CMRR (dB)	66	− *	70	87
PSRR (dB)	75	−50.5	63	44
Supply voltage (V)	2.8	±1.5	1.8	±1.5
Offset (mV)	− *	45	− *	0.01

* Data not available in the literature.
